# Impact of Critical Material Attributes (CMAs)-Particle Shape on Miniature Pharmaceutical Unit Operations

**DOI:** 10.1208/s12249-020-01915-6

**Published:** 2021-03-11

**Authors:** Mohammad A. Azad, Gerard Capellades, Allison B. Wang, David M. Klee, Gregory Hammersmith, Kersten Rapp, David Brancazio, Allan S. Myerson

**Affiliations:** 1 Department of Chemical Engineering, Massachusetts Institute of Technology, 77 Massachusetts Avenue, Cambridge, MA 02139 USA; 2 Present Address: Department of Chemical, Biological and Bioengineering, North Carolina A& T State University, 1601 E. Market Street, Greensboro, NC 27411 USA; 3 Present Address: Department of Chemical Engineering, Henry M. Rowan College of Engineering, Rowan University, 201 Mullica Hill Road, Glassboro, NJ 08028 USA; 4 Present Address: On Demand Pharmaceuticals, 3 Park Ave, 33rd floor, New York, NY 10016 USA

**Keywords:** critical material attributes (CMAs), needle-shaped particles, miniature pharmaceutical unit operations, Ciprofloxacin HCl, direct compressible tablets

## Abstract

The U.S. Food and Drug Administration (FDA) emphasizes drug product development by Quality by Design (QbD). Critical material attributes (CMAs) are a QbD element that has an impact on pharmaceutical operations and product quality. Pharmaceutical drugs often crystallize as needle-shaped (a CMA) particles and affect the process due to poor flowability, low bulk density, and high compressibility, and eventually the product performance. In this study, the product obtained from crystallization was needle-shaped Ciprofloxacin HCl (CIPRO), formed lumps during drying, and compacted during processing through feeders. To delump small amounts of materials and break the needles, multiple available devices (mortar-pestle, Krups grinder) and custom-made grinder were assessed before formulation. The processed CIPRO powder was then used to make tablets in the miniature tablet manufacturing unit developed by the team at MIT. The critical quality attributes (CQA) of the tablets, set by the United States Pharmacopeia (USP), were then assessed for the drug powder processed with each of these devices. Powder properties comparable to commercial CIPRO were obtained when the custom MIT-designed grinder was used, leading to tablets that meet the USP criteria, with comparable dissolution profiles of those for marketed CIPRO tablets. This study demonstrates how needle-shaped crystals have an impact on pharmaceutical operations, even if it is on a miniature scale, and how proper shape and subsequent flow properties can be obtained by processing the particles through the MIT team-designed grinder.

**Supplementary Information:**

The online version contains supplementary material available at 10.1208/s12249-020-01915-6.

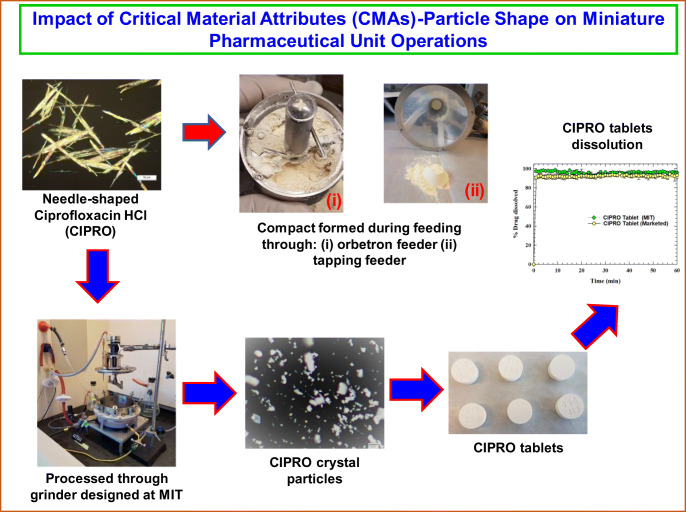

## Introduction

The U.S. Food and Drug Administration (FDA) encourages the adoption of quality by design (QbD) principles in the development and manufacturing of drug products (1). FDA emphasizes quality must be built into the manufactured product. QbD elements include the critical quality attributes (CQAs) of the drug product, identification of critical material attributes (CMAs) and critical process parameters (CPPs), and linking CMAs and CPPs to CQAs (1). A CMA is a physical, chemical, microbiological, or biological characteristic or property of an input material that should be within an acceptable range, limit, or distribution to ensure the anticipated quality of that in-process material, excipient, or drug substance (1). CMAs can significantly impact pharmaceutical unit operations, process consistency, and product quality attributes (2). Hence, material properties need to be tested and CMAs need to be defined and controlled.

Pharmaceutical drugs or active pharmaceutical ingredients (APIs) often crystallize as particles with high aspect ratio, typically as needle-shaped particles or elongated plates (3). This behavior has been reported for salicylic acid, aliskiren hemifumarate, alisertib sodium, or melitracen hydrochloride, among others (4–7). Needle-shaped crystals, in particular, are typified by aspect ratios in the range of (≈ 1:1:100−1000) (8). However, it is worth noting that crystals with aspect ratios of (1:1:≳ 10) may still be “troublesome” during processing operations (8). Drug substance’s physical properties such as particle morphology (shape) have a significant impact when designing and developing a robust drug product having envisioned CQAs (1). The manifestation of needle-shaped API is often considered high risk and a source of difficulty (flowability tends to be minimized, which in turn can negatively impact processing characteristics such as hopper discharge, die filling, and other volumetric dosing operations) in the manufacture of pharmaceutical dosage forms (9). Drug particle shape can affect downstream processability and cause physical characterization difficulties and drug product performance issues such as a poor dissolution rate or bioavailability (9, 10). Particle shape can affect content and dose uniformity, the grittiness of solid particles in chewable tablets, and other properties related to physicochemical stability (11). Crystal shape and facet-specific mechanical properties can affect the fracture cleavage behavior of organic crystalline materials (12). Particles with less spherical shape tend to have larger contact area due to more uniform particle arrangements (13). This results in higher compact strength. Particle cohesiveness, to some extent, is desirable in the formulation process and for increased tablet strength; however, increased particle cohesiveness can lead to operational difficulties due to compaction of particles inside the process (13). The buildup of compacted material leads to an operational problem in constant uniform feeding.

In the pharmaceutical industry, opportunities to modify existing processes are often limited due to regulatory and safety requirements (8). Typically several strategies have been considered to handle needle-shaped particle formation: alteration to crystallization process using crystal habit modifying additives, manipulation of solvents, ultrasonication, and mechanical operation (milling) are some examples (14). Each approach has its drawbacks. For example, micronization via dry milling yields an increase in the cohesiveness of particles generates a risk for dust explosion, and it is unfeasible for stress-sensitive compounds and crystal forms (14, 15). Crystal habit modifying polymers can radically affect API solubility, resulting in unacceptable yields. Hence, its concentration must be controlled in the same way as any other impurity (9). Solvent selection is also limited due to toxicity, impact on environment, and yield (16). Given these limitations, milling and micronization are still, at present, an attractive approach routinely used to obtain consistent particle size and shape modification during downstream formulation processing (9, 12).

**Fig. 1:**
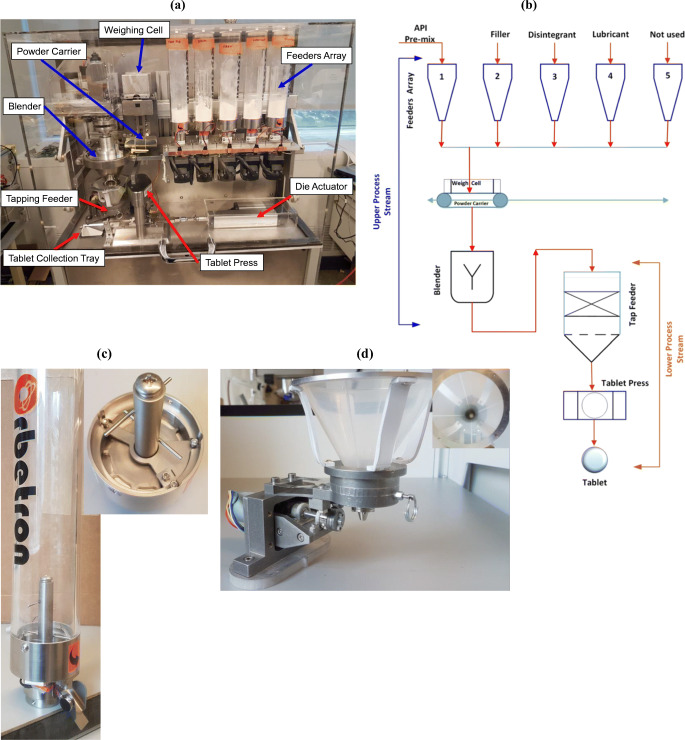
(**a**) Compact, portable, re-configurable, and automated tablet manufacturing unit [72.4 cm (length) × 53.3 cm (width) × 134.6 cm (height)], volume 0.52 m^3^. (**b**) Schematic of the process for direct compression tablet manufacturing. (**c**) Feeder to feed API and excipients. (**d**) Custom-designed tapping feeder to feed powder blend

At the Massachusetts Institute of Technology (MIT), we developed a compact, portable, re-configurable, and automated system (Fig. 1) that offers the means for on-site, on-demand manufacturing of pharmaceutical tablets from drug/API crystals (17, 18). The system was designed to achieve 1000 drug doses per day for immediate or close-to-immediate use. The system consists of the feeding of API and excipients, blending, dispensing, and compression to make tablets. It handles a few grams of materials as the number of doses is small compared to commercial manufacturing. In this study, we emphasize how particle shape, especially needle-shaped, critical material attributes, impacted on different unit operations of the system, even at a small scale. We also discuss different grinding options that were investigated to improve the processability of needle-shaped particles. Finally, we demonstrate that the grinder recently designed by the MIT team (19, 20) performed well for small-scale powder, that the powder can easily be processed through the system, and that the manufactured oral dosage tablets showed a comparable performance of the commercially marketed product.

## Materials and Methods

### Materials

Ciprofloxacin hydrochloride monohydrate (CIPRO, 99.8% purity) was purchased from Jai Radhe Sales (Gujrat, India) and used as the model drug. Bulk density, compression, biopharmaceutical properties, stability/target shelf life, *etc.*, determines the selection of excipients to design the drug product. Stability improvement excipients were not considered as manufacturing is an on-demand basis. A simplified method in formulation development was deliberated by reducing the number of excipients necessary for tableting (17, 21). Hence, to formulate only one filler/diluent, flow aid/glidant, and lubricant were chosen. Silicified microcrystalline cellulose (Prosolv SMCC® HD 90) was gifted by JRS Pharma GMBH & CO. KG (Rosenberg, Germany) and considered as the filler/diluent, fumed silica (CAB-O-SIL® M-5P) was gifted by Cabot Corporation (MA, USA) and considered as the Glidant, and Magnesium stearate (Kosher Passover HyQual™), gifted by Mallinckrodt Pharmaceuticals (MO, USA), was considered as the lubricant. Sodium starch glycolate (EXPLOTAB®) gifted by JRS Pharma GMBH & CO. KG (Rosenberg, Germany) was used as a disintegrant to improve the disintegration of CIPRO tablets, as this process yields a volume dosage form that requires a higher compaction force than the reported earlier work with the drugs used in the unit (17). SSG also improves subsequent dissolution (22, 23). The materials considered in this study, along with their particular roles and relevant physical properties, are presented in Table I.

**Table I:** Materials Used for Making CIPRO Tablets and Their Properties

Name of ingredient	Quantity	Percentage composition ((%) w/w)	Function	Particle size (μm)			Bulk density (g/cm^3^)	Compressibility (%)	ffc^*3*^
	mg			*d*_10_	*d*_50_	*d*_90_			
Ciprofloxacin HCl (CIPRO)^*1*^	291.00^*2*^	58.20	Active pharmaceutical ingredient	-	-	-	-	-	-
Silicified microcrystalline cellulose	189.00	37.80	Diluent	38.83	127.61	324.56	0.52	6.17	> 10
Fumed silica	5.00	1.00	Glidant/flow aid	-	-	-	-	-	-
Magnesium stearate	5.00	1.00	Lubricant	2.76	6.86	15.10	0.31	38.97	5.18
Sodium starch glycolate	10.00	2.00	Disintegrant	23.62	45.67	80.27	0.68	19.0	> 10
Total	500.0	100.0							

^*1*^Monohydrate

^*2*^Amount reflects conversion from monohydrate salt form to the free base (250 mg)

^*3*^Flow function coefficient

### Crystallization and Mechanical Processing of CIPRO

**Fig. 2:**
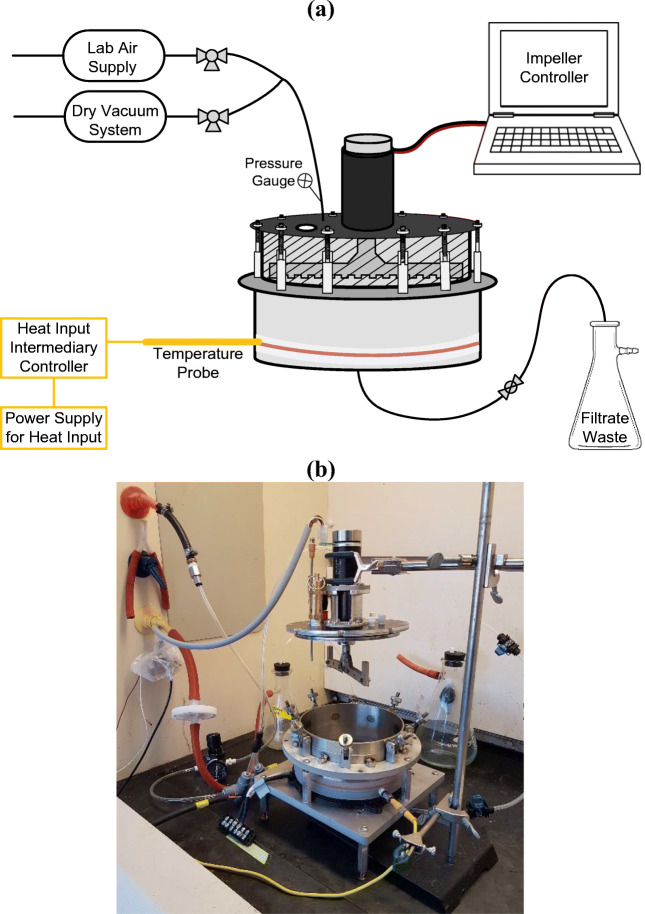
(**a**) Schematic of the grinder designed at MIT. (**b**) Picture of the grinder

The downstream process crystallized CIPRO as needle-shaped particles, with an aspect ratio of approximately 1:1:20. To obtain a similar particle shape from commercial material, the commercial CIPRO was recrystallized following the procedure that was intended for downstream manufacturing. The crystallization feed solution was prepared by dissolving 100 mg/mL of commercial Ciprofloxacin HCl in 25 vol% formic acid, 75 vol% water. Then, the API was recrystallized through the addition of isopropanol as an antisolvent. This was done in a continuous flow manner, as reported in previous work (24). The final antisolvent content was 80 vol%, yielding a suspension with approximately 18 mg/mL of crystalline API. Ultrasonication of the crystallized API was considered as an alternative for particle breakage of the needle-shaped crystals. For those tests, 150 mL of the crystallization suspension was sonicated using a Hielscher UP100H device (100 watts, 30 kHz) connected to an MS1 sonotrode, inserted directly into the sample. The sonication time was kept at 10 min, using a constant amplitude of 100%. Both the crystallized suspension and the samples processed by ultrasonication were filtered, washed with a 20 vol% water, 80% acetone solution, and dried at 50°C at 0.5 bar in a vacuum oven for 24 h. Industrial drying processes relying on Nutsche filter dryers tend to produce a large amount of lumps, even with agitated drying. Consequently, it is common to implement delumping processes before formulation. However, no suitable commercial equipment was found to delump the small amount of powder that is generated in this type of study. Hence, a mortar-pestle, a Krups grinder (Figure S1), and a customized grinder (Fig. 2) designed at MIT were used. The custom-built grinder is a compact device designed for the integrated filtration, drying, and mechanical processing of APIs. Details of the device can be found in Capellades et al. (19) and a patent application (20). In this study, the grinding experiments were conducted following the procedure mentioned in Capellades et al. (19). After 65 min of static drying, the powder was mechanically processed through agitated drying for 55 min, with a constant impeller rotation speed of 30 rpm. The powder properties are achieved in the agitated drying step by utilizing an impeller geometry that promotes delumping, controlling the distance between the impeller blade and the bottom of the plate at 0.5 mm, and using an impeller rotation program with direction switch as described in Capellades et al. (19). The delumped CIPRO was sieved using a 600-μm sieve (Figure S2) because the head of the blend dispensing unit (tapping feeder) has an opening hole of 700 μm. The obtained powder was then used for on-demand tablet manufacturing in the compact system.

### Physical Characterization of CIPRO, Excipients, and Blends

To develop and manufacture pharmaceutical formulations as well as finished products, it is important to characterize and understand API, excipients, and blend properties at the particle and bulk level (25). Bulk density, flow properties, size, and shape of CIPRO, excipients (explicitly fillers), and blends were assessed to develop the formulation. Scirocco 2000, dry powder dispersion unit of Mastersizer 2000 particle size analyzer (Malvern Panalytical Inc.), was used to measure the particle size of CIPRO and excipients. API or excipients were placed in an enclosed vibratory tray. Then, compressed air was used to suspend the particles of the sample and pass through the air cell. To feed the powder, a 50% feed rate setting was used in the vibratory tray of Scirocco 2000. A 2 bar air pressure was used to disperse the powder through the air cell. The Mastersizer 2000 provided volume-weighted particle sizes. The fumed silica CAB-O-SIL® M-5P has a median size of about 14 nm as reported elsewhere (26). Optical microscopy images of the CIPRO powder were captured with a Nikon microscope (Eclipse ME600) equipped with a Nikon DS-Ri1 camera.

FT4 Powder Rheometer (Freeman Technology, Tewkesbury, UK) was used to measure bulk density and powder flow properties of CIPRO, excipients, and blends. In the FT4, the bulk density test was done using conditioned bulk density test and completed before any other test. Shear cell test was used to measure powder flow property. Powder compressibility was also measured. Bulk density is a key factor in determining the amount of powder that can fit in a space such as a tablet press hopper, a tablet die, or a blender (25). Powder flow measurements and a flow coefficient can be obtained from the shear test. The flow function coefficient (ffc) is used as an indicator of the powder flowability and is defined as the ratio of the major principal stress to the unconfined yield strength. The ffc has been acquired in this work using 3 kPa of normal stress. Based on Schulze (27), ffc values can be defined into different regimes: ffc < 1, not flowing; 1< ffc < 2, very cohesive; 2 < ffc < 4, cohesive; 4 < ffc <10, easy flowing, and ffc > 10, free-flowing. Powder compressibility (the capability of powder to reduce in volume under pressure), denoted as the percentage change in volume after compression, was also measured. Compressibility does not directly measure the flowability; however, a free-flowing powder generally has a low compressibility value. A compressibility value of more than 30% specifies poor powder flow (28). The detailed procedures of each FT4 Powder Rheometer test can be found in the literature (29).

### Manufacturing and Characterization of CIPRO Tablets

CIPRO tablets were manufactured in the unit using the delumped and ground CIPRO powder. An 11-mm die and punch (round-shaped) set was used. One thousand-kilogram compression force was applied. Tablet properties such as weight, content uniformity, assay, tensile strength, and dissolution performance were evaluated. USP39-NF34 official monographs were followed to do all characterization (30).

### Tablet Weight and Tensile Strength

Tablet weight was determined in the weighing station (custom-designed) fabricated on a Sartorius load cell (Sartorius WZA 224-L). Tablet breaking force or hardness was measured using the tablet hardness tester (Dr. Schleuniger Pharmatron Model 6D). Tensile strength was calculated using tablet hardness according to the relationship mentioned in Azad et al. (21). This tensile strength was measured for six tablets and the average was reported.

### Assay of Tablets

Assay of the CIPRO tablets was conducted using high-performance liquid chromatography Ascentis® 25 cm × 4.6 mm × 5 μm C8 column and an Agilent 1200 series G1315D Diode Array Detector. For all the measurements, the column temperature was maintained at 30 ± 1°C and the detection wavelength at 278 nm. Ten microliters was injected to the mobile phase flowing at a rate of 1.5 mL/min. The mobile phase was set at a volumetric ratio of acetonitrile to solution C of 13:87. Solution C was 0.025 M phosphoric acid, adjusted with triethylamine to a final pH of 3.0 ± 0.1. The sample solution was prepared using five tablets in solution B using a 500-mL volumetric flask. The solution was filtered through a 0.45-μm filter and diluted to prepare the equivalent of the API concentration of 0.2 mg/mL. Solution B was prepared at a volumetric ratio of acetonitrile to solution A of 13:87. Solution A was 0.025 M phosphoric acid, having a pH of 2.0 ± 0.1 adjusted by triethylamine.

### Tablet Content Uniformity Determination

To confirm consistency, each tablet in a manufactured batch should have a drug substance content within 85–115% of the labeled content which is measured as 100%. Content uniformity or weight variation (USP-39 < 905 > Uniformity of Dosage Units) is the method that is considered to demonstrate uniformity (31). Uncoated tablets having 25 mg or more drug substance comprising 25% or more, by weight of the tablet, required to follow the weight variation method. The content uniformity method needs to be followed where the weight variation method does not meet the requirements. In this study, CIPRO was tested by the weight variation method. Ten CIPRO tablets were assayed using the assay methods described above. USP < 905 > Uniformity of Dosage Units method was used to calculate acceptance value (AV). AV of 15 or less should be expected to confirm content uniformity.

### Dissolution of Tablets

USP II paddle method using a Varian VK 7025 dissolution apparatus (Varian, Inc., USA) was used to perform tablet dissolution. USP 39–NF 34 official monograph was chosen to select the dissolution conditions. For CIPRO, the dissolution medium volume was 900 mL 0.1 N hydrochloric acid. The rotational speed of the paddle was 50 rpm. The temperature was kept at 37°C ± 0.2°C. In the beginning, tablets were manually added to the dissolution media. The UV was measured in an automatic Varian UV–Vis Cary 50 apparatus having the *in situ* probes set at 276 nm for CIPRO. A total of three tablet’s dissolution was measured. The average value with standard deviation was stated. The dissolution was compared with commercially available CIPRO tablets packaged by American Health Packaging (Columbus, OH 43217).

## Results and Discussion

### Particle Shape and Its Impact on Miniature Pharmaceutical Unit Operations

Figure 1a shows the tablet manufacturing unit (17, 21) and different miniature pharmaceutical unit operations such as different feeders for API and excipients, blender, tapping feeder for dispensing powder blend, tablet press, *etc*. Each component is a plug-and-play type and easy to assemble and disassemble. An enclosure was used for the solids module to prevent airflow that may cause weighing cell fluctuation and the transfer of dust powder (if any generates) to the environment. Figure 1b shows the process flow diagram (PFD), which represents the general flow of the process and equipment. There are five volumetric feeders (Orbetron 50 series micro feeder, OD50SV) mounted in the feeder array. In this study, four feeders were used. Each feeder consists of feeder housing, disc (50 mm in diameter, 10 pockets open hole), powder discharge chute having adafruit vibrating mini motor disc, and hopper (Fig. 1c). To improve cohesive powder flow, 18 g of 5-mm glass beads was added in the feeder and SiO2 was premixed with the drug powder. The beads help to enhance the powder flow by breaking the powder’s compaction when the disc rotates. All feeders feed the materials on the powder carrier, which transfers the powder into the blender. The blended powder is then fed into the tablet die using the custom-designed tapping feeder (Fig. 1d). The amount of materials used for blending is based on the percentage composition given in Table I and the total blend mass is 45.69 g.

**Fig. 3:**
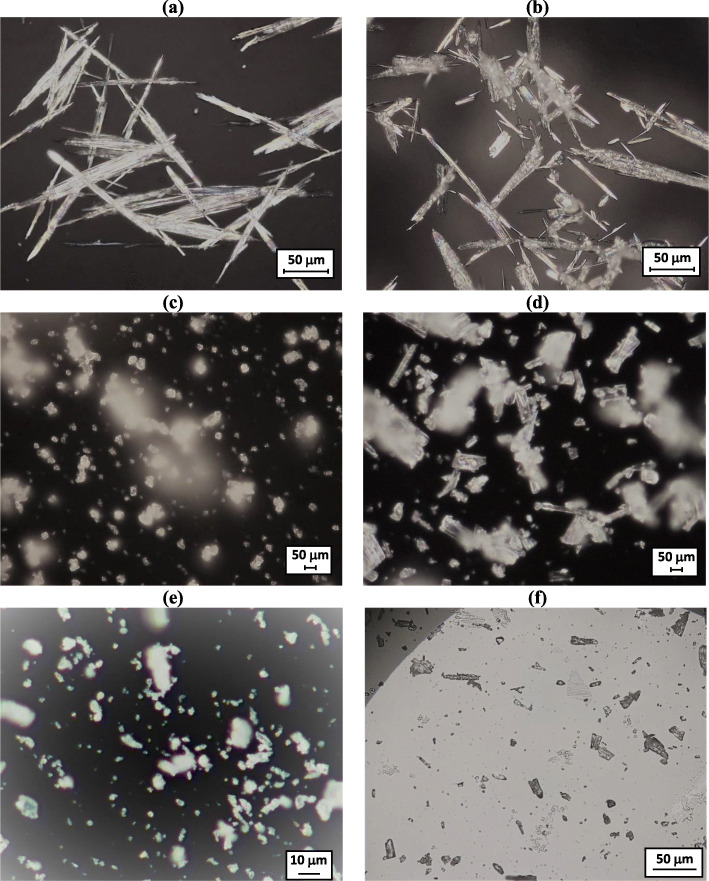
Optical microscopy image of Ciprofloxacin HCl (CIPRO) crystal particles: (**a**) downstream process crystallized (w/o ultrasound) needle-shaped CIPRO; (**b**) crystalized w/ ultrasound; (**c**) crystallized w/ ultrasound and ground w/ Krups grinder; (**d**) crystallized w/ ultrasound and ground w/ mortar and pestle; (**e**) crystalized w/o ultrasound and ground w/ MIT-designed grinder; and (**f**) commercially available and un-processed

Powder flow properties such as size, shape, and bulk density impacted the feeder performance. Table I shows material compositions that are used for making CIPRO tablets and their flow properties. The crystallized CIPRO was needle-shaped with a high aspect ratio (approximately 1:1:20) (Fig. 3a), and it was very fluffy. Hence, it is not suitable to use through the unit and its flow properties were not measured. Similarly, nano-silica, CAB-O-SIL® M-5P, flow properties were not measured as it is very fluffy due to its nano size, formed aggregates, and showed a lack of consistency in flow behavior. The bulk density of CAB-O-SIL® M-5P is < 0.06 g/cm^3^, reported by Cabot Corporation (32). The low bulk density value confirms the observation of fluffy behavior. Comparing the flow properties of all excipients, magnesium stearate has a finer particle size, low bulk density, high compressibility, and low ffc. In general, when particle size increases, powder flowability increases, a weaker interparticle force between particles is observed, and the cohesivity of the powder decreases (33). The powder bulk density increases as powder pack in a denser state due to an increase in particle size (33). Based on Schulze classification (27), both Prosolv SMCC® and EXPLOTAB® are free-flowing, whereas magnesium stearate flows easily. Hence, all excipient powders were easily handled through the feeders.

**Fig. 4:**
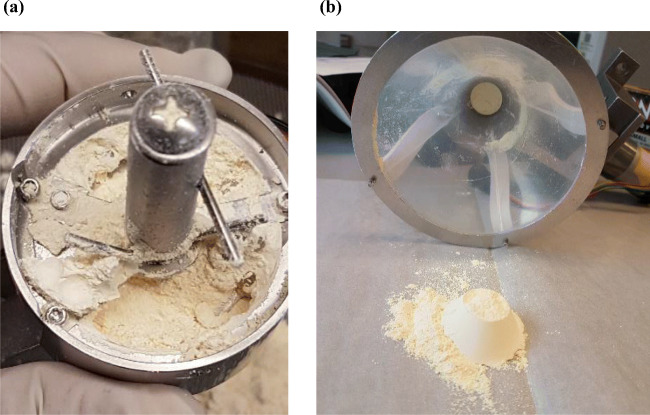
Processing issues observed due to needle-shaped CIPRO. Hard compacts formed during (**a**) CIPRO (premix with SiO2) feeding through Orbetron feeder and (**b**) CIPRO blend feeding through tapping feeder

As expected for elongated particles, the needle-shaped CIPRO occupies a large volume per unit mass. Its bulk density, as well as flow behavior, can be improved by reducing its aspect ratio. At first, ultrasound was applied to the crystallization suspension to attempt at breaking the needle-shaped crystals. While some effect was observed, sonication alone was unable to eliminate completely all particles’ needle shapes (Fig. 3b). Since there was no sufficient improvement in the crystals’ aspect ratio, CIPRO was now crystallized without ultrasound and delumped-grounded using the Krups grinder (Figure S1a, S2). The CIPRO premixed with CAB-O-SIL was then processed through the Orbetron feeder. However, the particles were not able to feed through the feeder after the first 1 or 2 min. They formed a compact mass which prevented the powder from flowing. Figure 4a shows the feeder after the powder flow stops. Since silica premixed CIPRO did not flow through the Orbetron feeder, it was added into the blender manually. The powder blend was prepared and dispensed into the tapping feeder. After a few taps of dispensing, the blend’s powder flow stopped due to the formation of a compact mass. Figure 4b shows the tapping feeder after the powder flow stopped. The silica premixed CIPRO powder and CIPRO blend behavior can be easily correlated with the flow properties found in FT4 measurements, as presented in Tables II and III. It is observed in Table II that CIPRO (w/o US, Krups Grinder) has a low bulk density (0.22 g/cm^3^) and ffc (1.26), and high compressibility (52.6). Similarly, the CIPRO blend (Table III) has a low bulk density and ffc, and high compressibility. Hence, CIPRO powder and blend had a very poor flow behavior. The overall findings show particle shape has an impact on pharmaceutical unit operations even if it is on a miniature scale.

**Table II:** Properties of Processed CIPRO Particles

API, excipients	Particle size (μm)			Bulk density (g/cm^3^)	Compressibility (%)	Flow function coefficient (ffc)
	*d*_10_	*d*_50_	*d*_90_			
CIPRO (w/o US*, Krups grinder)	1.23	3.88	33.26	0.22	52.6	1.26
CIPRO (US and Krups grinder)	1.93	7.09	65.36	0.38	38.8	1.56
CIPRO (US and mortar-pestle)	3.01	8.67	24.02	0.41	43.6	2.04
CIPRO (w/o US, grinder designed at MIT)	2.82	14.45	169.73	0.48	29.5	4.17
CIPRO (commercially available, un-processed)	3.75	12.49	439.56	0.39	38.9	2.95

*US: ultrasound applied during crystallization

**Table III:** Properties of Powder Blends Prepared for Tableting

Blends	Bulk density (g/cm^3^)	Compressibility (%)	Flow function coefficient (ffc)
CIPRO (w/o US*, Krups grinder)	0.44	32.5	2.06
CIPRO (US & Krups grinder)	0.55	23.0	2.41
CIPRO (US & mortar-pestle)	0.51	23.2	4.67
CIPRO (w/o US, grinder designed at MIT)	0.57	12.4	>10
CIPRO (commercial, un-processed)	0.69	21.1	8.25

*US ultrasound applied during crystallization

### Processing of Needle-shaped CIPRO Particles

Only applying ultrasound during crystallization or using Krups grinder for crystallized CIPRO did not help to break the needle shape of CIPRO particles and subsequently improve powder flow properties. Hence, the combined process was applied. For this, ultrasound was applied during crystallization, and the dried powder was subsequently ground. CIPRO was ground using either Krups grinder or mortar and pestle (Figure S1a, b). Mortar and pestle can handle a small mass of materials and it can apply gentle shear. However, its operation is biased due to human handling, and it is not efficient for handling a large amount of powder. Krups grinder can handle a large amount of CIPRO but it works at one speed. It also applies high stress compared to a mortar and pestle. Both mortar and pestle and Krups grinder helped delumping. The properties of CIPRO powder crystallized using ultrasound and ground using Krups or mortar and pestle are given in Table II. No appreciable difference in flow properties was observed when comparing to CIPRO particles ground with Krups grinder with those ground with a mortar and pestle. The bulk density and compressibility were also in the same range. The optical microscopy images of CIPRO particles (Fig. 3c) show that very fine particles were produced for the Krups grinder, whereas for mortar and pestle, the particle size was slightly reduced (Fig. 3d). However, it was not possible to eliminate the needle-shaped crystals completely even after applying the combined process. Comparing to Krups ground particles, mortar and pestle ground particles have higher aspect ratios (see Fig. 3c, d). This is due to low shear applied to particles by the mortar and pestle. The comparison of powder blends of both types of blend shows no significant difference in flow properties (Table III).

The combined approach of ultrasonication and off-line mechanical processing was not able to improve powder flow properties. Therefore, the powder was not suitable for dispensing and tableting. As an alternative approach, we attempted to integrate the mechanical processing of the API within the drying step in drug substance isolation. In this context, we designed and developed a miniature unitfor integrated filtration, drying, and mechanical processing of APIs (19, 20). This unit would allow us to conduct necessary drug substance isolation processes, and utilize a combination of mechanical processing and *in situ* feedback control on the API dry matter content to eliminate the needle-like crystals during the drying process. Figure 2 (a, b) shows a schematic and the grinder itself, respectively. CIPRO powder produced using the MIT-designed device shows a high bulk density (0.48) and ffc (4.17), and low compressibility (29.5). The desirable ffc value is greater than 4 and a compressibility value of less than 30% (27). Comparing all processed powders, it has better powder flow properties (see Table II), and it does not require the addition of off-line mechanical processing between drug substance isolation and formulation. It is also noted that the CIPRO used for the MIT grinder was crystallized without any ultrasound applied in the downstream process. The particle size *d*_5__0_ and *d*_90_ are larger for the MIT-designed grinder compared to other processed particles. The particle size (specifically *d*_50_:14.45 *vs* 12.49 μm) and flow properties are similar to the commercial CIPRO powder manufactured in bulk and purchased from the market. Commercial CIPRO powder did not require any processing, and it flows very well through the tablet manufacturing unit in Fig. 1. The particle shape was observed in optical microscopy and shown in Fig. 3e. There were no more long needle-shaped particles detected. Instead, a combination of fine and large particles was observed with aspect ratios around or below 1:1:2, similar to what is observed for commercially processed CIPRO. The particle shape of commercially processed CIPRO is shown in Fig. 3f. This corroborates the particle size distribution presented in Table II. The powder blend was also prepared and characterized by FT4. Results show that the blend properties were significantly improved for the CIPRO processed in the MIT-designed device. The silica premixed CIPRO powder and CIPRO blend were processed through the Orbetron feeder and tapping feeder. There were no issues observed. Both the powder and blend flowed well.

### CIPRO Tablet Manufacturing and Performance Evaluation

**Fig. 5:**
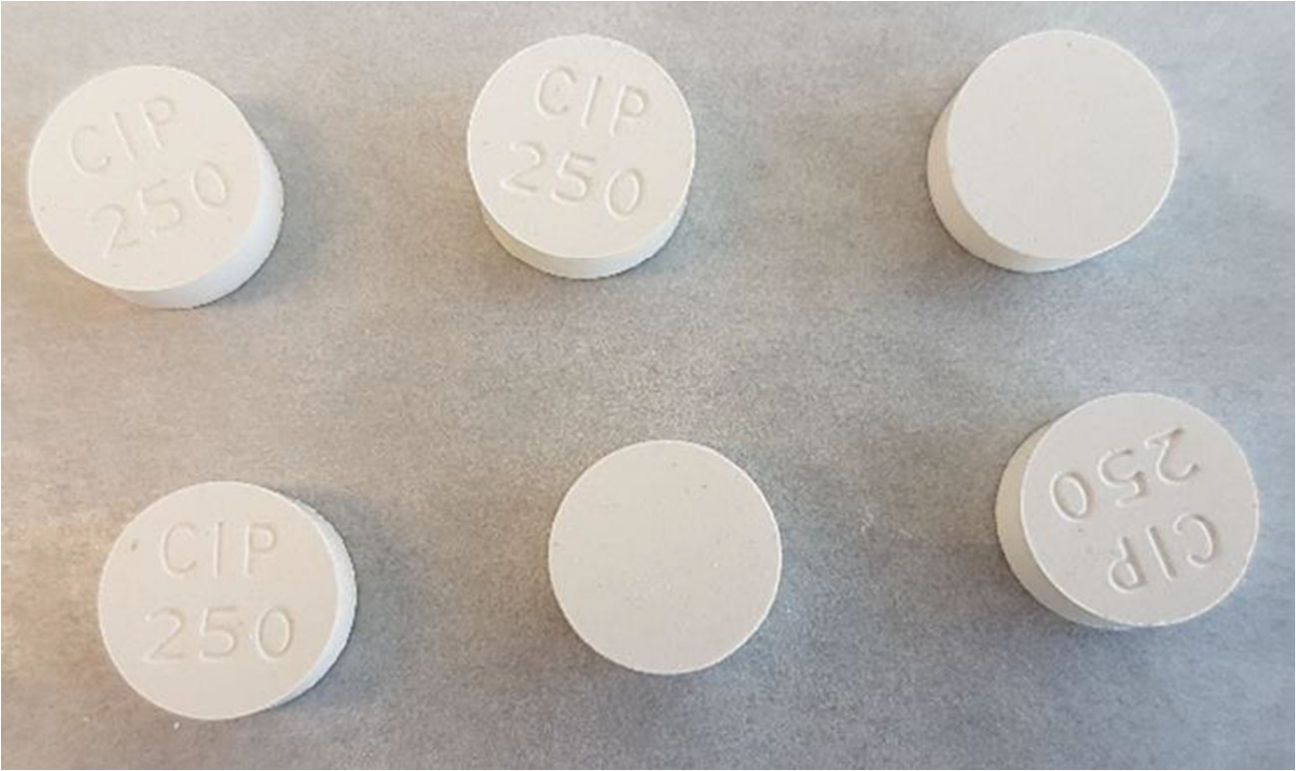
Picture of CIPRO tablets produced

To make directly compressible tablets, the powder should have good flow properties, be readily compressed, and have a high bulk density. Large variability in tablet weight, poor content uniformity, and inconsistent tablet properties such as breaking force, dissolution, and disintegration can occur if the powder has poor flow (34). Powder blend produced using CIPRO from the MIT-designed grinder shows good flow properties, so tablets were manufactured using the miniature manufacturing unit. Figure 5 shows a picture of those tablets. Following the intended dosage of 250 mg of CIPRO, tablets are embossed with the text “CIP 250”. Ten tablets average weight with %RSD, as presented in Table IV, was 479 mg, and the weight variation was within 10% of the target weight. The RSD value is 5%, which indicates a uniform blend is being dispensed into the die, and the tablets manufactured in the unit have low weight variations. The average diameter, thickness, and tensile strength of six tablets are 11.08 mm, 3.88 mm, and 4.67 MPa, respectively. A small increase in the tablet diameter was observed as a result of gradual radial recovery during ejection. Seton et al. (35) reported an observation of gradual radial recovery of tablets. Tensile strength is an essential quality attribute that impacts post compaction process operations such as dissolution, coating, storage, and handling. The tablet strength depends on the formulation composition and compression force applied. In this work, the maximum compression force 1000 kg was applied by the tablet press.

**Table IV:** Properties of Tablets (Weight, Tensile Strength, Assay, and Acceptance Value) Prepared Using Blends

Blends	Tablet weight (Average, % RSD) (mg)	Tensile strength (average ± STDEV) (MPa)	Assay (% of the labeled content) (%)	Acceptance value (AV)
CIPRO (w/o US, grinder designed at MIT)	479.0, 5.0	4.67 ± 0.09	97.42	12.95

*RSD*: Relative Standard Deviation, *STDEV*: Standard Deviation

**Fig. 6:**
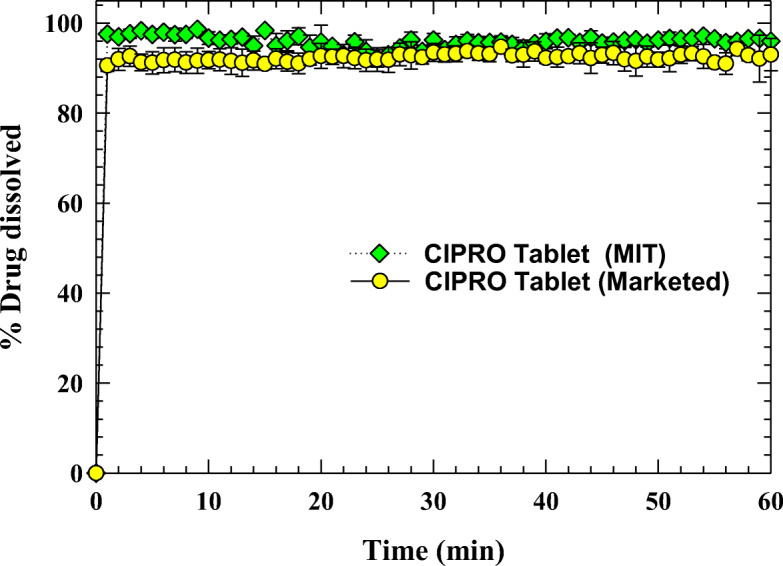
Comparison of the dissolution of CIPRO tablets manufactured at MIT with commercially available tablets in a USP II apparatus. Average and standard deviation are depicted (*n* = 3)

According to the USP-39 official monograph, tablets meet the assay standard when tablets are between 90 and 110% of the labeled amount of API. In the same way, the tablet meets the USP quality standard if the calculated acceptance value of CIPRO is less than or equal to 15.0, based on 10 dosage units. It is observed from Table IV that the assay value of CIPRO tablets is within the range, 90~100%, and that it meets content uniformity/weight variation criteria since the acceptance value (AV) is lower than 15. Figure 6 shows the evolution of the dissolution of CIPRO tablets after manufacturing and compared with commercially available tablets in a USP II apparatus. According to the USP monograph, 85% of the CIPRO should dissolve within 30 min. The CIPRO tablets meet the USP monograph. The faster dissolution of CIPRO tablets implies instant release tablets, caused by a faster disintegration due to the presence of super disintegrant SSG. The dissolution profile is comparable to the profile for commercial tablets. Initially, the commercial tablets have slow-release compare to MIT-manufactured tablets that may be due to the coating of the commercial tablets.

## Conclusions

Particle shape can impact powder flow behavior. Pharmaceutical unit operations are sensitive to particle shape, especially for systems presenting high aspect ratios. Grinding is very well-known in the pharmaceutical industry. However, to handle a small amount of powder, there is no miniature equipment available commercially. At MIT, we designed a grinder integrated with a filter dryer that produces dry CIPRO powder with optimum flow properties which are suitable for tableting. The tablet properties meet USP criteria and the dissolution profile is comparable to commercial CIPRO tablets. The overall findings, comparing this system to common alternatives, demonstrate that needle shape has an impact on pharmaceutical processing even on a miniature scale. These challenges have been overcome by the MIT-designed grinder.

## Supplementary Information

ESM 1 (PDF 303 kb)
