# Supplementary material for: Impact of Critical Material Attributes (CMAs)-Particle Shape on Miniature Pharmaceutical Unit Operations
Source: AAPS PharmSciTech. 2021 Mar 11;22(3):98. doi: 10.1208/s12249-020-01915-6 (PMC7952360; doi:10.1208/s12249-020-01915-6)
Supplement: Supplementary file 1 — (PDF 303 kb) [file 12249_2020_1915_MOESM1_ESM.pdf]

<u>**Supplementary Material**</u>

Impact of Critical Material Attributes (CMAs)-Particle Shape on Miniature Pharmaceutical Unit Operations

Mohammad A. Azad[1], Gerard Capellades,[2] Allison B. Wang, David M. Klee, Gregory Hammersmith[3], Kersten Rapp[3], David Brancazio, Allan S. Myerson[4]

Department of Chemical Engineering, Massachusetts Institute of Technology, 77 Massachusetts Avenue, Cambridge, MA 02139, USA

1. Currently at Department of Chemical, Biological and Bioengineering, North Carolina A& T State University, 1601 E. Market Street, Greensboro, NC 27411, USA

2. Currently at Department of Chemical Engineering, Henry M. Rowan College of Engineering, Rowan University, 201 Mullica Hill Road, Glassboro, NJ, 08028, USA

3. Currently at On Demand Pharmaceuticals, 3 Park Ave, 33rd floor, New York, NY 10016, USA.

4. Corresponding author

Phone: 617-452-3790

Email: myerson@mit.edu

a) (b)

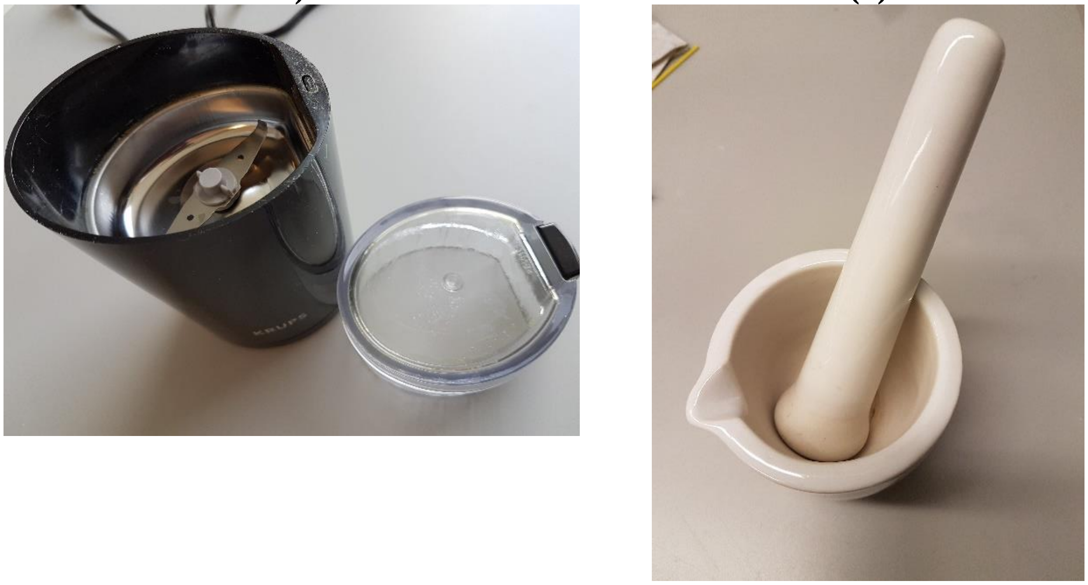

Figure S1. Azad et al. (2020), intended for *AAPS PharmSciTech*.

Picture of equipment used for delumping-grinding of CIPRO: (a) Krups grinder; (b) Mortar-Pestle.

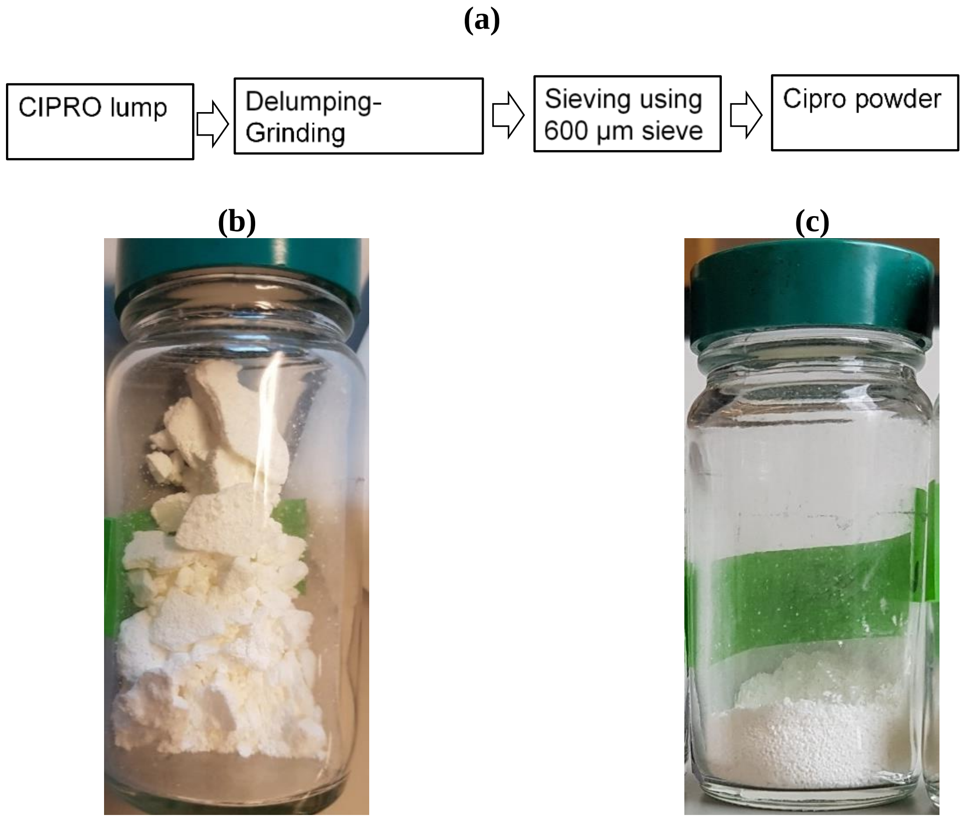

Figure S2. Azad et al. (2020), intended for *AAPS PharmSciTech*.

(a) Schematic of processing steps followed to produce Ciprofloxacin HCl (CIPRO) powder from the lump; (b) lump from the downstream unit after filtration and drying; (c) powder after delumping-grinding and sieving.
